# A Modified Trilinear Post-Cracking Model for Fiber-Reinforced Concrete to Improve the Evaluation of the Serviceability Limit State Performance

**DOI:** 10.3390/ma18071395

**Published:** 2025-03-21

**Authors:** Fan Zhang, Wouter De Corte, Xian Liu, Yihai Bao, Luc Taerwe

**Affiliations:** 1 College of Civil Engineering, Tongji University, 1239 Siping Road, Shanghai 200092, China; fanzhang@tongji.edu.cn (F.Z.); xian.liu@tongji.edu.cn (X.L.); luc.taerwe@ugent.be (L.T.); 2 Department of Structural Engineering and Building Materials, Ghent University, Technologiepark Zwijnaarde 60, 9052 Ghent, Belgium; 3 State Key Laboratory for Hazard Reduction in Civil Engineering, Tongji University, Shanghai 200092, China; 4 Department of Civil and Systems Engineering, Johns Hopkins University, Baltimore, MD 21218, USA; ybao11@jh.edu

**Keywords:** fiber reinforced concrete, constitutive model, fiber pull-out, three-point bending test, FEM, parameter analysis

## Abstract

An accurate constitutive model for fiber-reinforced concrete (FRC) is fundamental for analyzing and designing FRC structures. The recently released *fib* Model Code 2020 (MC2020) includes significant modifications to the tensile constitutive model for FRC, enhancing its accuracy. However, it has been observed that the applicability of this model for certain types of FRC is limited due to its overly simplified post-cracking mechanical assumptions. This is particularly evident in structural FRC, where the fiber pull-out force reaches its maximum at a large fiber slip, resulting in a load decrease before increasing again after the notched beam cracks. In that case, the bilinear assumption in the stress–strain model of MC2020 for post-cracking is insufficient to reflect the fiber mechanism and the mechanical properties of FRC at small crack widths. Therefore, based on the characteristics of fiber pull-out in structural FRC, this paper proposes a trilinear post-cracking stress–strain model to reflect the fiber pull-out mechanism more accurately and better analyze the performance of FRC structures in the serviceability limit state. Through an analysis of experimental data and numerical simulation studies on steel fiber-reinforced concrete (SFRC) notched beams, the parameters for the proposed trilinear constitutive model are determined and validated, and the results indicate that the stress value at the new inflection point in the post-cracking trilinear model should be 0.8*f*_Fts_ (the serviceability residual strength of the FRC). Although the proposed trilinear model seems similar to the trilinear model provided in MC2020, it is developed based on fiber pull-out behavior, whereas the trilinear model in MC2020 was mainly developed to eliminate numerical singularities. Finally, while the models in MC2020 perform well in evaluating the ultimate limit state performance, the proposed constitutive model can serve as a supplement, especially when serviceability limit state performance is considered.

## 1. Introduction

Compared to plain concrete, fiber-reinforced concrete (FRC) has better crack control capabilities and can partially replace steel reinforcement. In recent years, it has been widely used in various engineering structures, such as industrial floors [1,2,3,4], slabs [5,6,7], and tunnel segments [8,9,10,11]. When designing FRC structures, an accurate constitutive model for FRC is essential, especially the tensile constitutive model, because fibers have a minimal impact on the compressive performance of concrete but can significantly alter the residual strength after cracking. Therefore, the tensile constitutive model for FRC is a key focus in the design of FRC structures.

Currently, there are two main methods for determining the constitutive model of FRC. The first method involves analyzing the interaction mechanism between individual fibers and concrete at the microscopic level, considering factors such as the fiber inclination angle and fiber quantity, to derive the tensile constitutive model for FRC with the target fiber content [12,13,14]. The advantage of this method is that it does not require extensive testing. By analyzing data from single fiber pull-out tests [15,16], the constitutive model for FRC with different fiber contents can be determined, and as such, the economic cost is relatively low. This method also provides a more delicate stress–strain (crack opening) constitutive model for FRC. However, it requires extensive mathematical derivation, demanding a high theoretical level from the designer. Additionally, when the fiber shape or concrete matrix is changed, the relevant formulas need to be re-derived, making the process cumbersome.

Another method involves conducting tensile or flexural tests on FRC specimens to obtain post-cracking parameters. Based on post-cracking mechanical assumptions, sectional analyses are performed on FRC specimens at characteristic crack widths to derive the tensile constitutive model for FRC. Relevant standards [17,18,19,20,21] provide testing methods for FRC and corresponding formulas for calculating constitutive parameters. The main advantage of this method is its simplicity, making it more accessible for designers. However, it requires extensive testing to determine post-cracking parameters, which is economically costly. Additionally, the constitutive model is simplified into linear or multi-linear models, leading to some degree of inaccuracy.

Due to its simplicity and directness, the second method is commonly used to determine the tensile constitutive model for FRC in structural design. Based on this method, Germany first proposed an FRC constitutive model in 2001 [17]. Subsequently, RILEM [18], the Italian standard [19], the Spanish Recommendation [20], and the *fib* Model Code 2010 (MC2010) [21] released their tensile constitutive models for FRC, respectively. Blanco et al. [22] provided a detailed summary of these FRC constitutive models. Given the international influence of *fib* and di Prisco’s [23] detailed argumentation, the FRC constitutive model in MC2010 has been widely adopted. For instance, Liao et al. [24] conducted a critical analysis of FRC segments based on ductility requirements and the model in MC2010, proposing a design method to prevent brittle failure of segments. Nogales et al. [25] used the FRC constitutive model in MC2010 for numerical simulations and a flexural design of reinforced FRC slabs. Barragán et al. [26] applied the model in MC2010 to design glass fiber-reinforced floors. Guanziroli et al. [27] used the model in MC2010 to design secondary tunnel linings.

Although the FRC constitutive model in MC2010 has greatly promoted research on and applications of FRC structures, its simplified post-cracking assumption limits its applicability. The residual tensile strength in the model is determined by only two special crack widths, with the residual tensile strength for other crack widths obtained through linear interpolation, leading to inaccuracy in the description of residual strength. Additionally, some parameters are derived only when the *f*_R3_/*f*_R1_ ratio (*f*_R1_ and *f*_R3_ are the flexural tensile strengths corresponding to crack widths of 0.5 mm and 2.5 mm in the three-point bending test, respectively) is 0.5, resulting in errors for other ratios. Therefore, in recent years, researchers have proposed more constitutive models for correction. For example, Woo et al. [28] and Galeote et al. [29] proposed a post-cracking trilinear constitutive model through inverse analysis to improve simulation accuracy. Vandevyvere et al. [30,31] adjusted the parameters of the MC2010 constitutive model based on different classifications of FRC.

After a decade of development, fib released the Model Code 2020 (MC2020) [32] in 2024. The new code updates the relevant content based on MC2010, with significant modifications to the FRC constitutive model, allowing the new model to more accurately reflect the mechanical properties of FRC. However, the simplification methods and basic assumptions of the MC2020 model remain unchanged from MC2010; thus, the model still has limitations in its application. Specifically, the linear assumption leads to inaccuracy in the residual strength at small crack widths, resulting in significant errors when simulating the performance at the serviceability limit state (SLS) of certain structural FRCs, such as with hooked-end fibers.

Therefore, this paper first analyzes how these parameters of the FRC constitutive model are obtained in MC2010 and MC2020, explaining the differences and reasons for updates between the two models. Then, combined with the fiber pull-out mechanism, the limitations of the current model’s application are highlighted, and a new post-cracking trilinear constitutive model is proposed to more accurately reflect the fiber mechanism under service conditions. Finally, the constitutive parameters of the proposed model are determined through the analysis of experimental data and numerical simulations. The proposed post-cracking trilinear constitutive model is similar in form to the one proposed in MC2020 but differs in the mechanical background, resulting in different parameters. This paper elucidates the sources of the constitutive parameters in MC2020 and analyzes the fiber mechanism at different stages, aiming to clarify the applicability of the constitutive model for designers, thereby improving accuracy when evaluating the mechanical properties of FRC structures.

## 2. An Overview of the FRC Constitutive Model in the *fib* Model Codes 2010 and 2020

### 2.1. An Overview of the Constitutive Models

**Figure 1:**
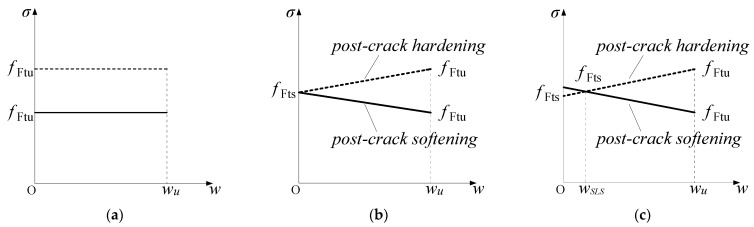
Schematic diagram of stress-crack opening laws in MC2010 and MC2020 (see Table 1 for parameters). (**a**) Rigid plastic model in both MC2010 and MC2020; (**b**) Linear model in MC2010; (**c**) Linear model in MC2020.

**Figure 2:**
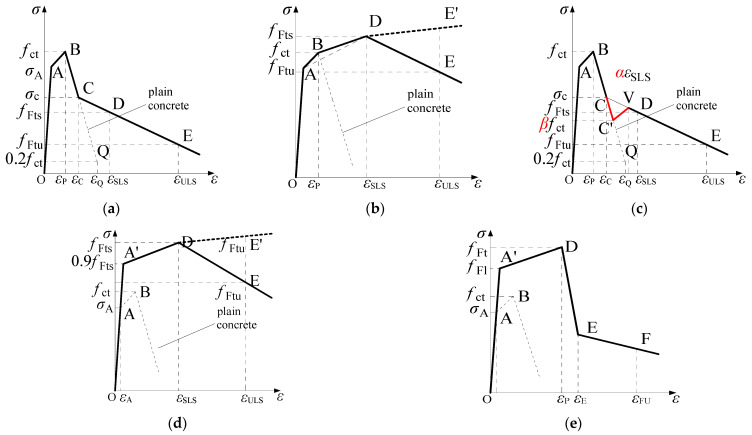
Schematic diagram of stress–strain laws in MC2010 and MC2020 (see Table 1 for parameters). (**a**) Post-cracking bilinear model for FRC showing softening behavior in both MC2010 and MC2020; (**b**) Post-cracking bilinear model for FRC showing hardening behavior in MC2010; (**c**) Post-cracking trilinear model for FRC when $f_{Fts}$ is larger than 0.8$f_{ct}$ in MC2020; (**d**) Model when FRC shows hardening pre-peak behavior in MC2010; (**e**) Model when FRC shows hardening pre-peak behavior in MC2020.

**Figure 3:**
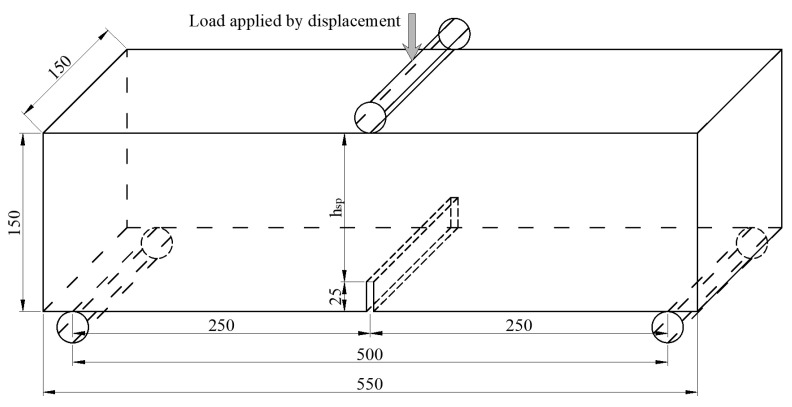
Schematic diagram of three-point bending test.

A comparison of the constitutive models in MC2010 and MC2020 is shown in Figure 1 and Figure 2 and Table 1. These models are derived based on the three-point bending test (Figure 3) recommended by EN 14651 [33]. A load–crack mouth opening displacement (CMOD) can be obtained, and the relevant stresses *f*_R1_ and *f*_R3_, when the CMOD is 0.5 mm and 2.5 mm, respectively, will be used to calculate the constitutive parameters. Both versions of the FRC constitutive models are divided into two main categories: the stress-crack opening model and the stress–strain model. The stress-crack opening model can only be used to evaluate the performance of FRC at the ultimate limit state (ULS). When it is necessary to analyze the performance of FRC structures in the SLS, the stress–strain constitutive model must be applied. The two versions of the constitutive models are generally similar but differ in the details, and the structural characteristic length (*l*_cs_) is used to connect the two types of models. The characteristic length is the “bridge” to connect continuous mechanics, governed by stress–strain constitutive relationships, and fracture mechanics, governed by a stress-crack opening law [23]; usually, it is the crack spacing, and the strain can be transferred to the crack width by multiplying it with the value of *l*_cs_.

In the stress-crack opening model, there are two types: rigid plastic and linear models. The rigid plastic models in MC2010 and MC2020 are the same, but the linear models differ in the calculation of the parameters for the serviceability residual strength (*f*_Fts_; corresponding strain, *ε*_SLS_) and the ultimate residual strength (*f*_Ftu_; corresponding strain, *ε*_ULS_). As shown in Table 1, compared to MC2010, the *f*_Fts_ value in MC2020 is smaller, and the new code specifies that the crack width corresponding to *f*_Fts_ is 0.5 mm. If the ULS crack width *w*_u_ is set to 2.5 mm, as stipulated in both codes, the difference in *f*_Ftu_ between MC2020 and MC2010 is 0.7*f*_R3_ − 0.6*f*_R1_, which is not significant. Since the stress calculation formulas also apply to the stress–strain constitutive model, these changes have the same influence on the stress–strain constitutive model. According to Table 1, No.x is used to represent the constitutive model in this paper, for example, No.1 represents the rigid plastic model.

In the stress–strain constitutive models, when FRC shows a significant post-cracking softening response, constitutive model No.3 in Table 1 should be used. This model is a post-cracking bilinear model: the stress first decreases along line BQ, which is the same as the plain concrete, and then changes along line DE, which is defined by the stress–strain states at the SLS and the ULS. The endpoint of the stress path along the plain concrete stress–strain curve is intersection point C of BQ and DE. In both new and old codes, apart from the aforementioned stress calculation parameters, all other calculations are the same.

When FRC shows a post-cracking hardening response, the constitutive model in MC2010 does not consider the reduction in stress after cracking. The stress linearly increases from the cracking point *f*_ct_ to the SLS point *f*_Fts_. However, in MC2020, the constitutive model accounts for the reduction in stress after cracking, reflecting the impact of crack localization at initial cracking. Additionally, the applicable condition changes to *f*_Fts_, being larger than 0.8*f*_ct_ instead of *f*_ct_. In this model, the endpoint of the stress decrease is no longer intersection point C of BQ and DE, but point C’ on BQ, where the stress is β*f*_ct_. The stress then linearly increases to point V, which is on line CDE, with a strain of αε_SLS_. According to MC2020 recommendations, the value of α is 1, and the value of β is 0.75. This post-cracking trilinear model is a new, unique constitutive model introduced in this update.

For conventional engineering, where the concrete class typically does not exceed C80 and the fiber content does not exceed 80 kg/m^3^, the above four types of constitutive models are generally suitable. For ultra-high-strength concrete and a higher fiber content, both the proportional limit strength and cracking strength will significantly increase, and constitutive models No.5 and No.6 in Table 1 should be used. The relevant differences are shown in Table 1. Overall, MC2020 emphasizes determining model parameters through uniaxial tensile test data and no longer uses the post-cracking residual strength calculation formula in No.2 as concrete usually exhibits multiple cracks, while the formula in No.2 was derived under the assumption of a single crack.

Additionally, MC2020 suggests that the constitutive model can be determined through inverse analysis rather than just using the above models when experimental data are available to retrieve more accurate constitutive models in numerical simulations.

### 2.2. Derivation and Applicability Analysis of Constitutive Models

The previous section introduced the types and parameters of the constitutive models. For FRC in MC 2010 and 2020, the derivation of the model parameters will be explained in this section to help readers gain a deeper understanding of the models and to apply these models correctly in practice. Since rigid plastic model No.1 is relatively coarse, and constitutive models No.5 and No.6 are not intended for conventional concrete and fiber contents, the derivation mainly focuses on models No.2 to No.4, and the derivation and equations in this section are valid only for beams with rectangular cross-sections.

#### 2.2.1. Serviceability Limit State

**Figure 4:**
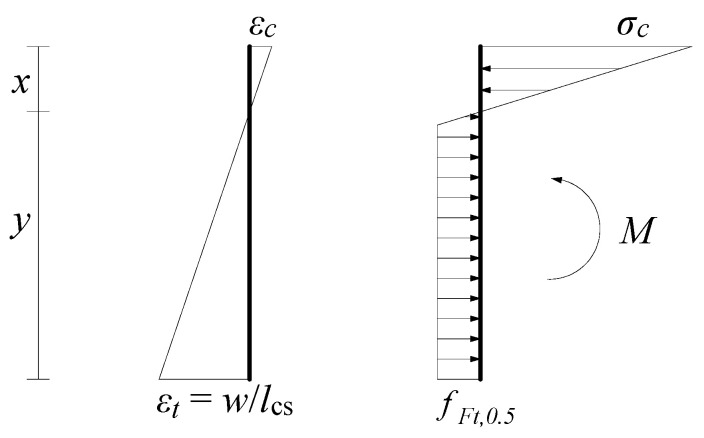
Stress and strain diagrams for FRC in the SLS.

The constitutive parameters of MC2010 and MC2020 are obtained from the cross-sectional analysis of three-point bending beams. When deriving the constitutive model parameters for the SLS, both MC2010 and MC2020 analyze the state when the crack width is 0.5 mm, and it is assumed that at this state, the tensile zone of FRC exhibits elasto-plastic behavior. This means it is linearly elastic until the tensile stress reaches *f*_Fts_, and then it is ideally plastic. Meanwhile, due to the relatively small compressive stress, the compressive zone is considered to be in a linear elastic state. This assumption is the same in both MC2010 and MC2020. The stress–strain relationship is shown in Figure 4.

Through analyzing the axial force and bending moment, Equations (1)–(3) are established. By solving these equations, the relationship between the residual strength *f*_Ft,0.5_ at a crack width of 0.5 mm and the residual strength *f*_R1_ can be obtained as shown in Equation (4). Specifically, *f*_Ft,0.5_ is *k*_a_ times *f*_R1_, where *k*_a_ is a function of the concrete elastic modulus *E* and the structural characteristic length *l*_cs_. This derivation has been detailed in [23] and will not be elaborated on here.

(1) $$\sigma_{c}=\frac{0.5Ex}{(h_{sp}-x)l_{cs}}$$

(2) $$\frac{\sigma_{c}bx}{2}-\frac{f_{Ft,0.5}^{2}bx}{2\sigma_{c}}-f_{Ft,0.5}b\left(h_{sp}-x-\frac{f_{Ft,0.5}}{\sigma_{c}}x\right)=0$$

(3) $$f_{Ft,0.5}b\left(h_{sp}-x\right)\left(\frac{2x}{3}+\frac{h_{sp}-x}{2}\right)-\frac{f_{Ft,0.5}^{2}bx}{2\sigma_{c}}\left(\frac{2x}{3}+\frac{f_{Ft,0.5}x}{3\sigma_{c}}\right)=\frac{f_{R1}bh_{sp}^{2}}{6}$$

(4) $$f_{Ft,0.5}=k_{a}(E,l_{cs})f_{R1}$$

In these equations, *σ*_c_ is the compressive stress at the edge of the compressive zone, *E* is the elastic modulus, *x* is the height of the compressive zone, *h*_sp_ is the height of the cracked section of the beam (measured from the top of the specimen to the tip of the notch), *l*_cs_ is the structural characteristic length, and *b* is the beam width.

In [21,23,32], *l*_cs_ is set to 125 mm, and this value has been demonstrated to be reasonable in [23]. Therefore, *k*_a_ is determined by the elastic modulus. Ref. [23] showed that when the elastic modulus varies from 20,000 MPa to 50,000 MPa, the difference in *k*_a_ is minimal, with an average value of 0.37 being adopted.

**Figure 5:**
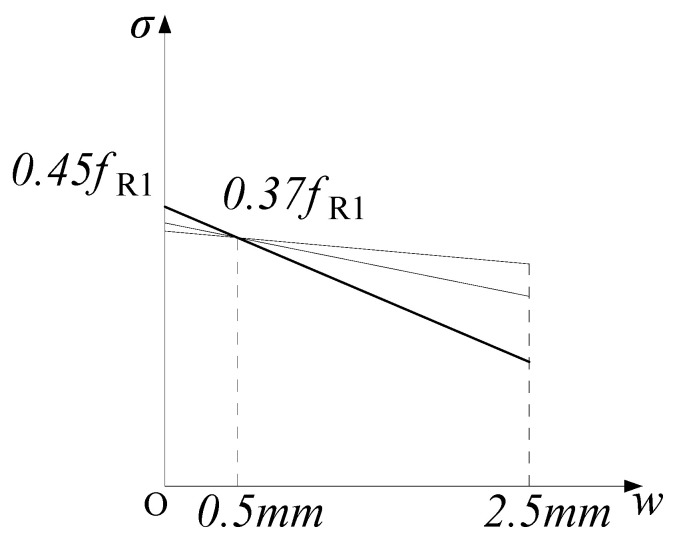
Stress overestimate when crack opening is smaller than 0.5 mm with *f*_Fts_ associated with crack opening of *w* = 0.5 mm.

When a post-cracking linear model is adopted, for FRC with the same *f*_Ft,0.5_ value, the smaller the *f*_Ftu_ value is, the larger the residual strength at crack widths less than 0.5 mm will be (Figure 5). This will result in unconservative strength and stiffness predictions when simulating FRC structures at crack widths less than 0.5 mm [23].

To avoid this phenomenon, ref. [23] suggests setting *f*_Fts_ as the residual strength at a crack width of 0 mm. Based on a linear assumption and assuming *f*_R3_ is 0.5*f*_R1_, the coefficient *k*_b_ between *f*_Fts_ and *f*_R1_ at a crack width of 0 mm can be derived as 0.45.

For constitutive model No.2, MC2010 adopted this suggestion, so in its constitutive model diagram, *f*_Fts_ corresponds to 0 mm rather than 0.5 mm, and *k*_b_ is adopted for calculation. However, for constitutive models No.3 and No.4, MC2010 sets the strain corresponding to *f*_Fts_ at a crack width of 0.5 mm, and the calculation coefficient remains 0.45. This results in the observation that the parameter does not conform to the derivation assumptions, leading to an overestimation of the residual strength of FRC when using the stress–strain constitutive model.

MC2020 corrected this inconsistency by setting *f*_Fts_ as the residual strength at a crack width of 0.5 mm in both stress-crack opening and stress–strain models, with a calculation coefficient of 0.37 (*k*_a_). However, as previously mentioned, due to the linear assumption, setting *f*_Fts_ at a crack width of 0.5 mm leads to an overestimation of the residual strength for crack widths less than 0.5 mm. Therefore, when using constitutive models No.2 and No.3 in MC2020 to analyze FRC structures with smaller cracks, the structural performance will appear better than it actually is.

The adjustment of the crack width corresponding to *f*_Fts_ is the fundamental reason for the difference in calculating *f*_Fts_ between MC2010 and MC2020.

#### 2.2.2. Ultimate Limit State

**Figure 6:**
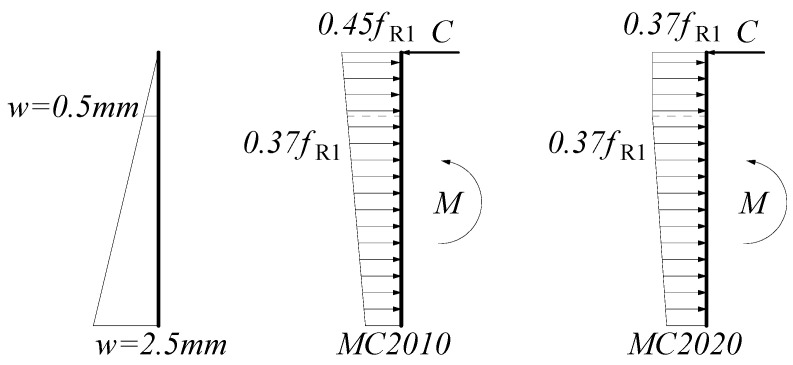
Stress and strain diagrams for FRC in ULS.

When deriving the constitutive model parameters for the ultimate limit state, both MC2010 and MC2020 analyze the state at a crack width of 2.5 mm. Due to the full development of cracks, the compressive zone is assumed to be only the upper edge of the beam. The crack opening and strain states for deriving the constitutive parameters in MC2010 and MC2020 are shown in Figure 6. MC2010 assumes a completely linear distribution under tensile stress. However, when deriving the constitutive parameters for the SLS, it is assumed that FRC shows ideal plastic behavior at a crack width of 0.5 mm. Therefore, in MC2020, the stress is corrected to show a bilinear distribution along the section, with the stress remaining constant for crack widths smaller than 0.5 mm.

By analyzing the bending moment, the stress distribution assumption in MC2010 results in Equation (5), which can be solved to obtain Equation (6). The stress distribution assumption in MC2020 results in Equation (7), which can be solved to obtain Equation (8). The above analysis is based on the state at a crack width of 2.5 mm. When the ULS crack width is set to be smaller than 2.5 mm, the stress is determined by linear interpolation, resulting in the calculation formula for *f*_Ftu_ in Table 1.

(5) $$f_{Ft,2.5}\frac{bh_{sp}^{2}}{2}+(0.45f_{R1}-f_{Ft,2.5})\frac{bh_{sp}^{2}}{6}=f_{R3}\frac{bh_{sp}^{2}}{6}$$

(6) $$f_{Ft,2.5}=0.5f_{R3}-0.225f_{R1}≅0.5f_{R3}-0.2f_{R1}$$

(7) $$f_{Ft,2.5}\frac{bh_{sp}^{2}}{2}+\left(0.37f_{R1}-f_{Ft,2.5}\right)\cdot \left(\frac{bh_{sp}^{2}}{50}+\frac{14bh_{sp}^{2}}{75}\right)=f_{R3}\frac{bh_{sp}^{2}}{6}$$

(8) $$f_{Ft,2.5}=0.5682f_{R3}-0.2607f_{R1}≅0.57f_{R3}-0.26f_{R1}$$

The difference in stress distribution assumptions is the fundamental reason for the difference in calculating *f*_Ftu_ between MC2010 and MC2020.

#### 2.2.3. Trilinear Post-Cracking Constitutive Model

The above-mentioned derivations explain the reasons for the differences between models No.2 and No.3 between MC2010 and MC2020. However, when the tensile strength *f*_ct_ of the FRC is similar to that of plain concrete but with a larger residual strength, constitutive model No.4 in Table 1 should be used. MC2010 proposes that when *f*_Fts_ is larger than *f*_ct_, the residual strength of the FRC increases linearly to *f*_Fts_ after cracking. However, when *f*_Fts_ is slightly lower than *f*_ct_, there may be no intersection point between lines DE and BQ if *f*_Ftu_ is sufficiently small. In other words, MC2010 cannot determine the constitutive law for this type of FRC regardless of whether model No.3 or No.4 is used. To avoid this phenomenon and consider the crack localization at the initial cracking of the FRC, MC2020 proposes that a trilinear post-cracking model should be used, where *f*_Fts_ is larger than 0.8*f*_ct_. In this model, the stress in the FRC decreases to 0.75*f*_ct_ after cracking and then linearly increases to *f*_Fts_.

Indeed, correcting this numerical singularity and considering crack localization are the main reasons for the differences in model No.4 in MC2010 and MC2020.

#### 2.2.4. The Influence of the Characteristic Length on the Constitutive Parameters

One of the important assumptions in the above derivations is that *l*_cs_ is set to be 125 mm. However, as seen from Equation (4), the adopted value of *l*_cs_ affects the value of *k*_a_. Or, when determining the constitutive model according to the parameters given in MC2020, different characteristic lengths adopted in simulations will lead to different results in the evaluation of structural mechanical performance.

Strain and crack width can be converted according to Equation (9). The characteristic length is typically crack spacing. In the notched beam 3PB tests, only one crack will appear, so the characteristic length was taken as 125 mm (the height of the cross-section) in the above derivation. Ref. [23] demonstrated the correctness of this value, deriving the stress–strain constitutive parameters in [21,32].

(9) $$w=l_{cs}\varepsilon$$

However, when using the finite element method (FEM) to analyze FRC structures, the characteristic length is usually related to the element size. For example, the characteristic length of hexahedral elements is typically the element length of the side. Therefore, the characteristic length in FEM models is no longer 125 mm, and when analyzing slabs or common beams, the characteristic length will be much less than 125 mm to ensure a sufficient number of elements.

**Figure 7:**
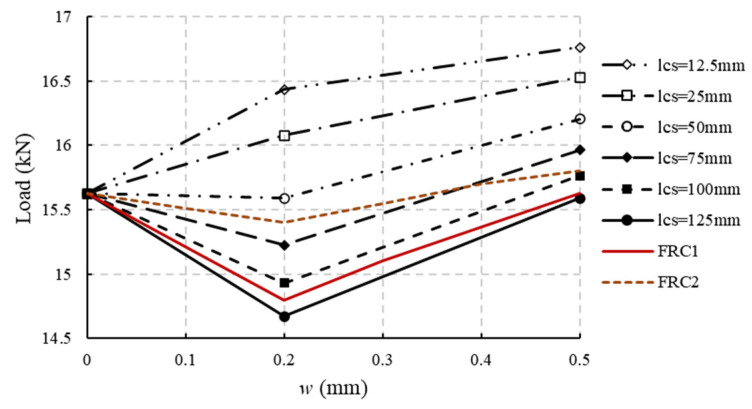
Load analysis for 3PB notched beam with different *l*_cs_ (parameter to convert strain to crack width in mm).

When the characteristic length becomes smaller, and if the constitutive parameters are still calculated according to the formulas in MC2020, this will result in an overestimation of the mechanical performance of FRC structures. Assuming an FRC with *f*_R1_ 5MPa, and that the cracking load is the same as the load at a crack width of 0.5 mm (uniaxial tensile strength of approximately 2.94 MPa), with an elastic modulus of 34,500 MPa, the load-bearing capacity of FRC beams for different characteristic lengths can be derived according to Equations (1)–(3), as shown in Figure 7. In Figure 7, FRC1 and FRC2 are selected as test result curves for two types of FRC (as the load–crack width curves can have similar trends even though the fiber content is different because of the unevenly distributed fibers, we select two curves to analyze). As shown in Figure 7, as the characteristic length decreases, the load-bearing capacity of the beam gradually increases. If the load-bearing capacity first decreases and then increases during the test (FRC1 in Figure 7), the simulation results with smaller characteristic lengths (smaller element size) will show greater errors at crack widths less than 0.5 mm. In Chinese standards [34], the crack width in the SLS for general structures is usually limited to 0.2 mm. Therefore, using the models in MC2020 to simulate FRC structures will overestimate the performance in SLS.

According to Equations (1)–(3), it can be calculated that when the elastic modulus is 34,500 MPa and the characteristic length decreases from 125 mm to 12.5 mm, the value of *k*_a_ decreases from 0.37 to 0.345, only showing a small change. However, if the influence of the characteristic length is not considered, the structural performance evaluation will be significantly affected. While the characteristic length is indeed a factor, it is not the fundamental reason for the inaccuracy in structural performance evaluation. As shown for FRC2 in Figure 7, when the characteristic length is 75 mm, the calculated results are closer to the actual results. The fundamental reason is that references [21,23,32] used a simple linear stress distribution assumption for the residual strengths. All post-cracking strengths are determined from only two cracking states, which is insufficient to reflect the complex mechanisms of how fibers strengthen concrete (named the fiber enhancement mechanism in later sections).

#### 2.2.5. Correspondence Between Constitutive Models and Fiber Enhancement Mechanisms

After concrete cracks, fibers enhance the concrete’s post-cracking behavior through bonding force, frictional force, and anchoring force between the concrete and fibers. The authors of [15] conducted single-fiber pull-out tests on hooked-end steel fibers and straight steel fibers to investigate fiber enhancement mechanisms.

**Figure 8:**
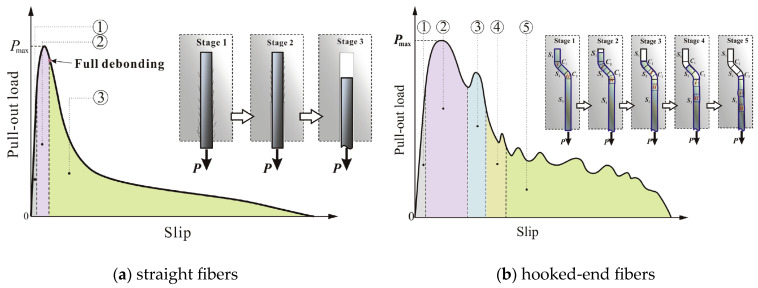
Schematic diagrams of pull-out behavior of straight and hooked-end fibers [15].

This study shows that the pull-out process of straight fibers can be divided into three stages (Figure 8a): the fully bonded stage, debonding stage, and the frictional sliding stage. In the fully bonded stage, the pull-out load increases linearly with slippage. As the load increases, interface damage occurs, and the fiber enters the debonding stage. During this stage, the load continues to increase, but the slip accelerates. The fiber reaches the maximum pull-out load in this stage. According to the test results in [15], the slippage is relatively small, about 0.2 mm, when the maximum pull-out load is reached. After complete debonding, the fiber enters the frictional sliding stage, where the pull-out load decreases rapidly but the slippage increases rapidly.

The pull-out process of hooked-end fibers can be divided into five stages (Figure 8b): the elastic and partial debonding stage, first plastic deformation stage, second plastic deformation stage, deformation–sliding hybrid stage, and frictional sliding stage. Due to the anchoring effect of the hooked end, the fiber slip remains small during partial debonding. In the first plastic deformation stage, the hooked end gradually deforms and is pulled out. As the load increases, the slippage accelerates. In this stage, the fiber reaches the maximum pull-out load, and the corresponding slippage is about 0.5 mm to 1.5 mm according to the test results in [15], which is much higher than that of straight fibers. In the subsequent three stages, the fiber is gradually pulled out, and the load decreases, but the decrease is slower compared to straight fibers. For more details, refer to [15].

Additionally, according to the pull-out study of fibers at different inclination angles in [35], when fibers are inclined, the load is lower for the same slippage. The fibers reach the maximum pull-out load at a greater slippage, and the maximum pull-out load is lower than that of vertically aligned fibers.

In FRC structures, fibers at the crack surface will exhibit various inclination angles (relative to the crack surface). The enhancement effect of fibers on the cracked structure is a comprehensive performance of the pull-out resistance of fibers at these different angles. Due to the multi-stage character of single-fiber pull-out, the post-cracking performance of the structure is complex. Although [15] analyzes the pull-out behavior based on two types of steel fibers, it can be extrapolated to two types of FRC structures. First, fibers reach the maximum resistance load and completely debond at a small slippage; in other words, the structural crack is small. After this, fibers cannot provide enough bridging load, causing the structural load-bearing capacity to continuously decrease as the crack grows. Second, fibers reach the maximum resistance load at a large slippage; in other words, the structural crack is large. Since the pull-out resistance of fibers is small at the initial stage when the crack is small, the structure will show a decrease in the load-bearing capacity as the crack develops. However, as the crack grows and the pull-out resistance of fibers increases to a sufficient level, fibers can provide enough resistance. Thus, as the crack develops, the structural load-bearing capacity gradually increases until most fibers reach their maximum pull-out resistance, after which the structural load-bearing capacity decreases again.

The post-cracking linear assumption in MC2020 [32] can be considered to correspond to the fiber enhancement mechanism of the first type of structure. However, for the second type of structure, due to the complexity of post-cracking behavior, the linear assumption cannot fully reflect the fiber mechanism. Additionally, MC2020 derives constitutive parameters using data at a crack width of 0.5 mm, where the pull-out resistance of fibers has already increased to a large level. If the linear assumption is used, it will overestimate the residual tensile strength of FRC at crack widths less than 0.5 mm, because at crack widths less than 0.5 mm, the fibers cannot provide larger pull-out resistance.

To address this issue, a post-cracking multi-linear model should be used to more accurately reflect the fiber mechanism. When the crack width exceeds 0.5 mm, the fibers usually reach their maximum pull-out resistance, or they reach the maximum pull-out resistance after a certain crack development and then gradually decrease. Therefore, using a linear model for crack widths greater than 0.5 mm will result in a conservative evaluation. However, the residual strength for crack widths larger than 0.5 mm is used to evaluate the ultimate load-bearing capacity of the structure, so a conservative result is reasonable. Thus, a linear model can still be used for crack widths larger than 0.5 mm. The residual strength for crack widths less than 0.5 mm is used to evaluate the performance of the structure in service states, so additional lines are needed to describe the fiber mechanism in this crack width range. Since the pull-out resistance of these fibers usually increases monotonically with the slippage for crack widths less than 0.5 mm, a bilinear model can be used for crack widths less than 0.5 mm, similar to constitutive model No.4 in MC2020. The first segment of the bilinear model for crack widths less than 0.5 mm represents a decrease in residual strength due to insufficient pull-out resistance of the fibers. The second segment represents the gradual increase in residual strength as the pull-out resistance of the fibers becomes sufficient and increases with the crack width.

It is important to note that although the proposed post-cracking trilinear model is formally similar to constitutive model No.4 in MC2020, its mechanical meaning is different. Model No.4 in MC2020 aims to eliminate potential numerical singularities and partially consider the crack localization effect at the initial cracking of concrete. Therefore, in this model, the stress at the new point, C’, is 0.75*f*_ct_, and the model’s application condition is that *f*_Fts_ is not less than 0.8*f*_ct_ (as numerical singularities are only likely under this condition). The proposed post-cracking trilinear model aims to more accurately reflect the fiber mechanism in post-cracking FRC. Thus, the model’s application condition is not evaluated based on the value of *f*_Fts_ but rather on the mechanical response of post-cracking performance: specifically, when the load decreases and then increases before the crack width reaches 0.5 mm or when fibers reach the maximum resistance load at a large slippage. Additionally, the stress at point C’ should reflect the post-cracking effect of the fibers, so it should be related to the residual strength rather than tensile strength. The most important issue for the new model is to determine the optimal stress at point C’, which can make the estimate of mechanical response weaker or stronger than the actual response if it is not suitable.

Deformed fibers, especially hooked-end steel fibers, can significantly enhance the post-cracking performance of concrete, making them widely used in engineering. Many test results indicate that FRC beams made by hooked-end fibers exhibit the aforementioned load decrease followed by an increase when making 3PBT. Therefore, determining the relationship between the stress at point C’ and the residual stress, and proposing a more suitable constitutive model for FRC made by deformed fibers, is important for accurately evaluating the SLS performance of FRC structures.

## 3. Analysis of Experimental Data

**Figure 9:**
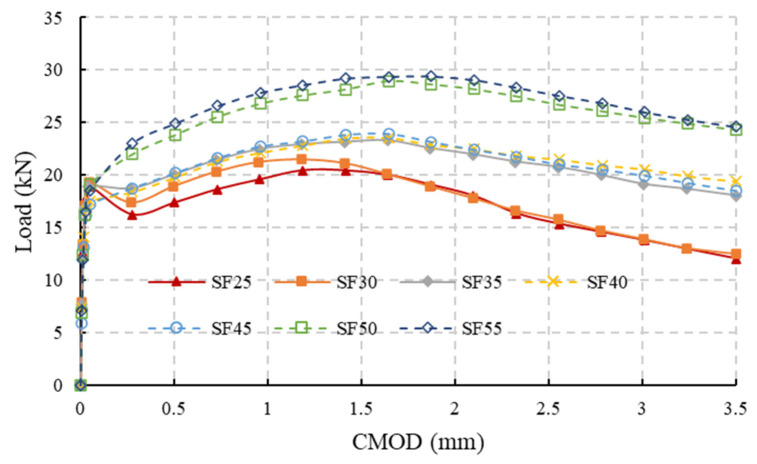
Three-point bending test results of notched beams with different steel fiber contents.

An analysis was conducted on the 3PBT results of notched beams made of SFRC with fiber contents ranging from 25 kg/m^3^ to 55 kg/m^3^ across seven groups. If the fiber content is less than 25 kg/m^3^, the FRC’s properties may not meet the requirements of MC2020 and it will rarely be used in engineering. On the other hand, if the fiber content is greater than 55 kg/m^3^, the FRC will not exhibit softening behavior after cracking, which is the topic of this paper, so fiber contents ranging from 25 kg/m^3^ to 55 kg/m^3^ are chosen. Each group consists of tests on 12 FRC beams, and the average results for each group are shown in Figure 9. The test results for different fiber contents are denoted as SFxx, where xx represents the fiber content. The details of the test can be found in [36,37]. As shown in Figure 9, when the fiber content reaches 45 kg/m^3^, the load does not significantly decrease after the concrete cracks. Therefore, the analysis first focuses on the test data for fiber contents lower than 45 kg/m^3^, totaling 48 test results. Because the fibers are not uniform in each specimen, some test groups with lower fiber contents also show no load decrease after cracking. These data are excluded. The test data of 35 specimens are selected for analysis.

Normally, when analyzing the test results of 3PBT of a notched beam, only the concrete cracking load F_LOP_ and the loads corresponding to crack mouth opening displacements (CMODs) of 0.5 mm, 1.5 mm, 2.5 mm, and 3.5 mm, denoted as F_1_, F_2_, F_3_, and F_4_, are focused on. However, to investigate the post-cracking trilinear constitutive parameters, it is necessary to consider the load changes before the crack reaches 0.5 mm. Therefore, the load F_min_, which is defined as the minimum load after concrete cracking and before the CMOD reaches 0.5 mm, is additionally extracted. These loads are shown in Table 2.

As the model code and EN 14651 do not give constitutive parameters related to F_min_, it is better to find the relation between F_min_ and F_1_, F_2_, F_3_, or F_4_ so that the new model can be proposed based on the frames of MC2020 and EN 14651, and no extra work should be performed when users use the proposed new model, such as the post-cracking trilinear model in MC2020, which connects the stress at C’ with *f*_ct_.

To investigate which load is most correlated with F_min_, a correlation analysis is conducted between F_min_ and the other characteristic loads. The calculated correlation coefficients between F_min_ and the other characteristic loads are shown in Table 3. As shown in the table, F_min_ has the strongest correlation with F_1_, with a coefficient of 0.7, and the weakest correlation with F_4_, with a coefficient of 0.42. Indeed, the correlation gradually decreases as the crack develops. This is because in the initial cracking stage, the bond and anchoring effects between the fibers and concrete primarily prevent crack development. In the later stages, the frictional force between the fibers and concrete primarily prevents crack development. Thus, as the crack develops, the fiber enhancement mechanisms change. When the crack widths are similar, the fiber enhancement mechanisms are also similar, resulting in stronger correlations between characteristic loads. On the other hand, the fiber enhancement mechanisms differ more as the crack grows, leading to a gradual decrease in correlation.

Additionally, the correlation between F_min_ and F_LOP_ is also relatively low, with a coefficient of 0.57, which is lower than its correlation with F_1_, and even with F_2_. Indeed, fibers do not significantly affect the tensile strength of concrete, resulting in a lower correlation.

**Figure 10:**
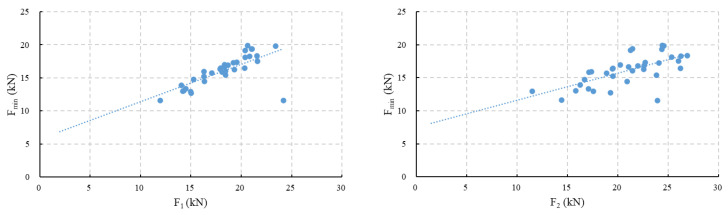
Fitting results of F_min_, F_1_, and F_min_, F_2_.

Fitting F_1_ and F_2_ with F_min_, as shown in Figure 10, indicates that F_min_ can be linearly represented by either F_1_ or F_2_. However, the scatter figure of F_min_ and F_2_ shows that the points are more dispersed. Although the correlation coefficients are close, the dispersion indicates that F_min_ has a closer relationship with F_1_.

The above analysis indicates that the minimum load after concrete cracking and before the CMOD reaches 0.5 mm can be represented by the load at 0.5 mm, F_1_. For the collected test set, the minimum load F_min_ is equal to 0.564F_1_ + 5.7873. Since *f*_Fts_ is linearly related to *f*_R1_, *f*_R1_ is linearly related to F_1_, and F_min_ is related to the stress at point C’, it can be inferred that the stress at point C’ is linearly related to *f*_Fts_. Determining the linear coefficient will determine the value of β in the proposed trilinear model.

## 4. Analysis of Constitutive Parameters

The optimal stress value at point C’ in the post-cracking trilinear constitutive model is unknown. Therefore, a parameter analysis of the stress value is conducted by FEM simulations.

In the finite element model, a crack band model is used to simulate the tensile cracking of FRC elements. The crack band model is chosen because it is developed based on continuous mechanics and is more efficient in calculation, and the influence of the mesh size on the results is reduced by introducing the crack bandwidth. When an element undergoes tensile cracking, the element strain consists of elastic strain *ε*^e^ and crack strain *ε*^f^ (Equation (10)). The elastic strain *ε*^e^ represents the strain in a cracked element still in the elastic stage, while the crack strain *ε*^f^ represents the strain caused by the crack. The crack width is calculated using Equation (11); therefore, in the finite element analysis, the post-cracking performance of FRC can be simulated by applying the stress-crack width constitutive model as we directly obtain the crack width from 3PBT, not the strain.

(10) $$\varepsilon =\varepsilon^{e}+\varepsilon^{f}$$

(11) $$w=l_{cs}\varepsilon^{f}$$

### 4.1. FEM Model

**Figure 11:**
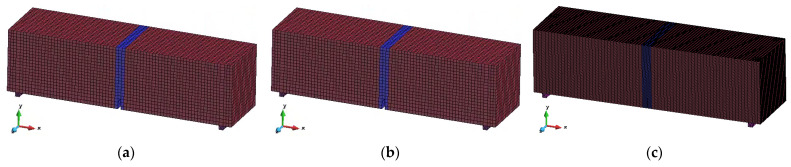
Diagrams of different numerical models. (**a**) element size 7.5 mm, fixed contact is adopted between beam and rollers; (**b**) element size 7.5 mm, slip contact is adopted between beam and rollers; (**c**) element size 2.5 mm, fixed contact is adopted between beam and rollers.

The FRC notched beam used to simulate 3PBT is established as shown in Figure 11. The dimensions of the notched beam in the model are 150 mm × 150 mm × 550 mm, with a notch depth of 25 mm at mid-span, the same as the test specimen (Figure 3). The notch width is set to 7.5 mm. Two steel rollers with dimensions of 10 mm × 10 mm × 150 mm are placed 250 mm from the mid-span to simulate the test roller.

In the model, the concrete beam is simulated using eight-node hexahedral elements, and the support roller is simulated using tetrahedral elements.

The notch width is around 2–3 mm in the tests. Therefore, it best matches the test conditions if the notch in the simulations is set to be 2–3 mm. However, this will cause more elements and make the simulations more time-consuming. To determine how the element size affects the simulation results, two models with different notch widths and mesh sizes are built: a notch width of 2.5 mm with an element size of 2.5 mm × 2.5 mm × 2.5 mm around the notch (far away from the notch, the element width is set to be 7.5 mm to reduce the number of elements), and a notch width of 7.5 mm with an element size of 7.5 mm × 7.5 mm × 7.5 mm. Only at the roller and below the notch, due to boundary size constraints, the mesh size slightly differs, but these are non-cracking elements and do not affect the simulation results. The simulations with a larger notch width are not considered as this is too far from reality.

Regarding the boundary conditions, a slip contact and a fixed contact between the beam and the rollers are considered and analyzed. When a slip contact is set, the friction coefficient is set to 0.1 as it represents contact between steel and concrete. Slip contact matches the real test condition better but makes the simulation more complex. If the fixed contact does not introduce significant errors, it is better to use fixed contact for further simulations.

During simulation, the translational movement in three directions of the left support roller is fixed, as well as the translational movement in the Y and Z directions of the right support roller, while the rotational degrees of freedom are not fixed. Displacement controlled load is applied by imposing a 2.5 mm displacement in the negative *Y*-axis direction over a 7.5 mm wide (or 2.5 mm if the notch width is 2.5 mm) surface, also at mid-span (not the whole top surface) to simulate loading, since the experimental 3PBT is operated by displacement control.

**Figure 12:**
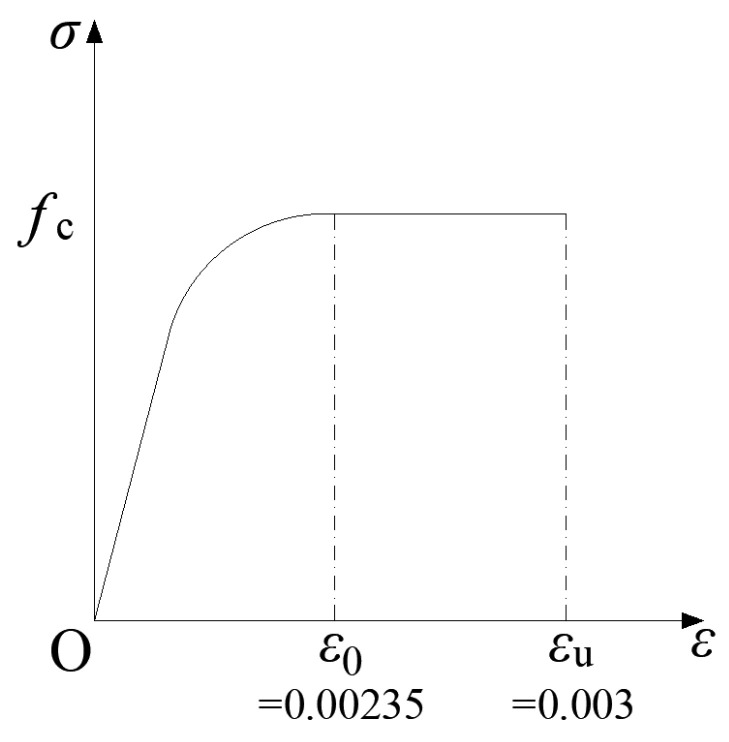
Diagram of compressive constitutive model.

Regarding material constitutive models, the rollers are simulated using an elastic model with an elastic modulus of 2 × 10^5^ MPa, while an elastic–plastic model is used for FRC. According to the test data, the compressive strength of the FRC is 77.54 MPa [36]. Referring to the “Code for Design of Concrete Structures” [34], the elastic modulus is set to 3.8 × 10^4^ MPa. In the test, the concrete does not undergo crushing failure, so the ductility of FRC does not affect the test results. Therefore, the compressive constitutive model of plain concrete in the “Code for Design of Concrete Structures” [34] is used to simulate FRC under compression (Figure 12). The tensile constitutive model adopts the proposed post-cracking trilinear model, which has the same form as model No.3 in Table 1, with the value of α set to 1, and the stress at point C’ is treated as a variable for parameter analysis. The tensile constitutive parameters are detailed in Section 4.2. The stresses and corresponding strains at each characteristic point in the model curve are input in the numerical software to define the FRC constitutive model.

### 4.2. Analysis Conditions

Since F_min_ shows good linear correlation with F_1_ when the fiber content ranges from 25 kg/m^3^ to 40 kg/m^3^, analyzing the constitutive parameters using the test data of any fiber content within this range does not affect the analysis results; therefore, since the test results for a fiber content of 25 kg/m^3^ show more occurrences of F_min_, the average values of this test group are first used for numerical simulation to determine the stress value at point C’. The relationship between this value and *f*_Fts_ is then determined to obtain the value of β.

In the test results [36,37], for a fiber content of 25 kg/m^3^, F_LOP_ is 19.1kN, and the proportional limit strength is calculated to be 6.11 MPa according to EN 14651 [33]. The proportional limit strength is approximately 1.7 times the tensile strength [38], so the tensile strength is 3.6 MPa. This is an approximate value as no direct tensile strength values are available, but it is acceptable and will not affect the mechanical response after cracking, which we are most concerned about. The average values of F1 and F3 are 17.35 kN and 15.51 kN [36,37], respectively. The values of *f*_Fts_ and *f*_Ftu_ in the constitutive model are calculated to be 2.057 MPa and 1.387 MPa [32,33], respectively. According to the post-cracking bilinear constitutive model in MC2020, the stress value at point C is 2.218 MPa, while for the proposed trilinear constitutive model, the value at point C’ should be smaller. By trying different stress values and analyzing the errors on the characteristic point loads, the optimal stress value at point C’ can be determined by finding the minimum error of F_min_ compared between the test and simulation results. The stress values at point C’ for the four trial calculations are shown in Table 4.

In addition, when analyzing the influence of the element size and the boundary conditions, only the bilinear model is used.

### 4.3. Simulation Results and Analysis

**Figure 13:**
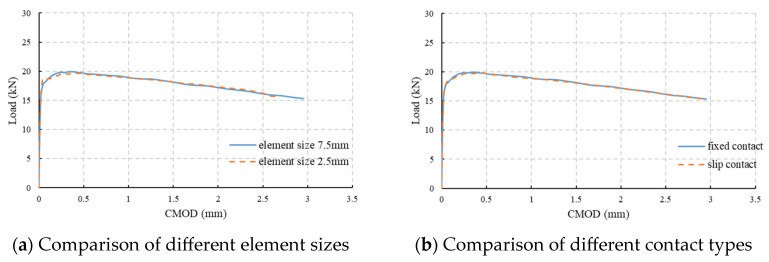
Analyses for different simulation conditions.

The simulation results with different element sizes and boundary conditions are shown in Figure 13. It can be observed that the simulation result with fixed contact and an element size of 7.5 mm is almost the same as those of the other two conditions, which means a slightly larger element size and simplified boundary condition can be accepted. Additionally, the simulation time for the fixed condition model is only half of that when slip contact is adopted, and for an element size of 7.5 mm, the calculation time is almost one-fifth of that when an element size of 2.5 mm is adopted. Therefore, the model with an element size of 7.5 mm and fixed contact will be adopted in further simulations.

**Figure 14:**
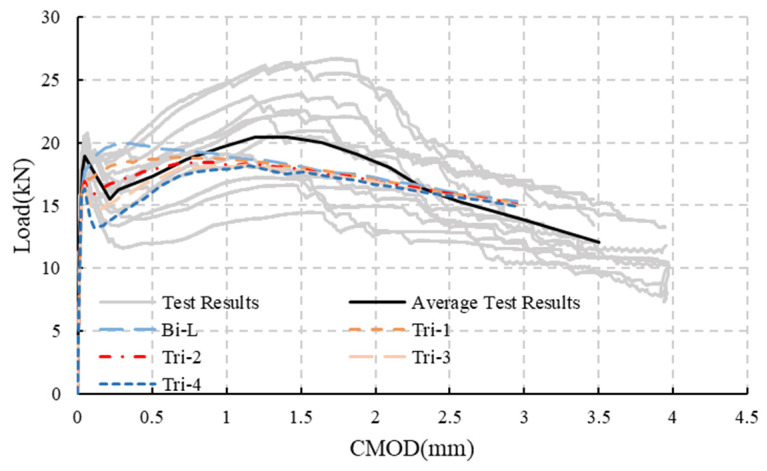
Simulation results of notched beams with different constitutive parameters.

The simulation results of bilinear and different trilinear models are shown in Figure 14. As shown in this figure, the load–CMOD curves obtained from simulations with different constitutive parameters are similar for CMODs larger than 1 mm, and the curves are generally in the middle range of the test result curves. This indicates that the different constitutive models do not affect the evaluation of the ultimate load-bearing capacity of FRC structures, and the evaluation results are reasonable. However, the different constitutive models have a significant impact on FRC beams before the crack reaches 1mm, especially before 0.5 mm.

When using the post-cracking bilinear constitutive model, the load does not decrease in the initial cracking stage, and it deviates significantly from the test results. When using the post-cracking trilinear constitutive model, the load decreases to varying degrees in the initial cracking stage, and the lower the stress value at point C’, the more obvious the load decrease. When the stress value decreases to 1.702 MPa, the load–CMOD curve shows a decrease followed by an increase, which aligns more closely with the test results.

The values of F_min_ in simulation results are shown in Table 5. The analysis indicates that F_min_ increases linearly with the stress value at point C’. Therefore, the optimal value of C’ is determined using linear interpolation. The average test value of F_min_ is 15.52 kN, so the optimal stress value at point C’ is calculated to be 1.639 MPa.

Since changes in the stress value at point C’ do not affect the load when the CMOD is larger than 1mm, the impact of the stress value at point C’ on F_min_ and F_1_ is analyzed. Additionally, a simulation with the optimal stress value at point C’ is included in the analysis. As shown in Table 5, when the stress value at point C’ minimizes the error of F_min_, the error of F_1_ is also the minimum one, further confirming the correctness of the optimal value.

Based on the analysis, it can be determined that the stress value at point C’ should be related to *f*_Fts_, and σ_c_’ should be equal to β*f*_Fts_. When *f*_Fts_ is 2.057 MPa, the optimal stress value at point C’ is 1.639 MPa; thus, the value of β is 0.8.

## 5. Verification of Constitutive Parameters

The above parameters were derived from test data with F_1_ = 17.35 kN (*f*_R1_ = 5.56 MPa). To further verify the applicability of the parameters, all 3PBTs shown in Table 2 were simulated. The results are shown in Appendix A. It can be seen that all the results show the same phenomenon: the curves obtained from the proposed trilinear model match the test curves better. Six sets of single-notched beam test data with *f*_R1_ ranging from 3.83 MPa to 7.67 MPa (representing different residual strength levels) were selected to conduct a detailed analysis. The differences between the numerical simulation results with different model parameters and the test results were compared. It should be noted that the selected six sets shown here are based on the classification rules of MC2020, where the FRC is classified according to *f*_R1_ (3–4 MPa, 4–5 MPa, 5–6 MPa, 6–7 MPa, and 7–8 MPa) and not based on the fiber content.

**Figure 15:**
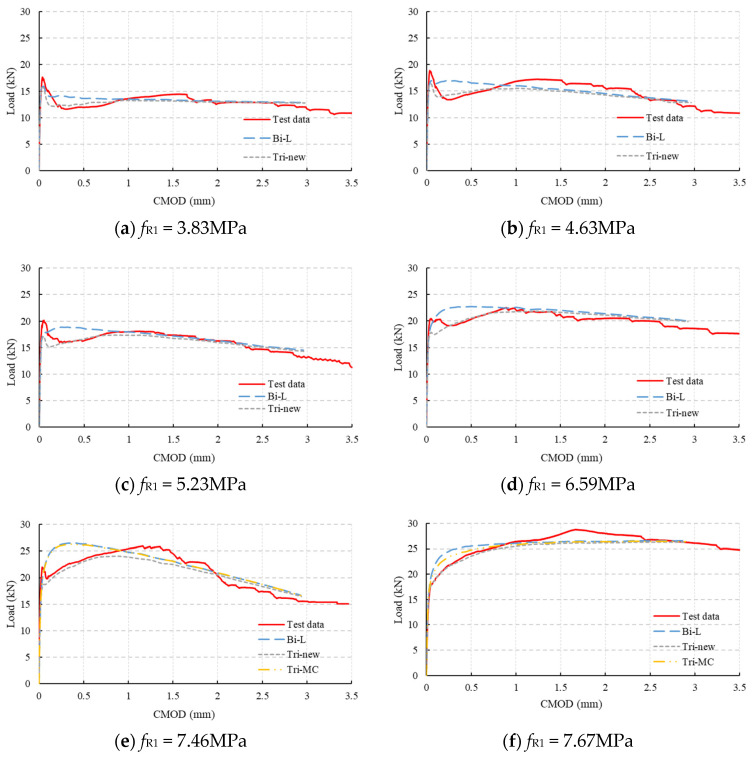
Comparison of simulation results through different constitutive laws for different SFRC classifications.

The simulation parameters are shown in Table 6 (*f^f^*_ct,L_ is the proportional limit strength in 3PBT). For Test 1 to Test 4, due to the low ratio of *f*_Fts_/*f*_ct_, only the simulation results of the post-cracking bilinear model (Bi-L) and the proposed post-cracking trilinear model (Tri-new) were compared. For the conditions of Test 5 and Test 6, the simulation results of the post-cracking trilinear model in MC2020 (Tri-MC) were also compared. The simulation results are shown in Figure 15.

As shown in Figure 15a–d, the post-cracking bilinear constitutive model in MC2020 results in higher loads before the crack reaches 1mm. In contrast, the load–CMOD curves obtained using the proposed post-cracking trilinear constitutive model closely match the test curves, indicating that the proposed model can more accurately simulate the mechanical response of FRC at small crack widths, and the proposed calculation parameter is correct.

As shown in Figure 15e,f, using the post-cracking trilinear constitutive model in MC2020 to simulate 3PBT results in load–CMOD curves that are slightly lower than those obtained using the bilinear constitutive model and closer to the test curves, but the loads are still high. In contrast, the simulation results obtained using the proposed post-cracking trilinear constitutive model closely match the test results. This is because the proposed model considers the fiber mechanism more accurately. Additionally, as shown in Figure 15f, when the load does not decrease after the initial cracking of FRC, the proposed model still accurately simulates the performance of the notched beam. This further indicates that the MC2020 constitutive model is overly simplified and not suitable to reflect mechanical performance at small crack states, making it unsuitable for investigating the SLS performance of structural FRC.

It is important to note that the fiber mechanism varies with the fiber shape, diameter, aspect ratio, fiber orientation, and concrete strength. Therefore, the parameters derived in this study may not be applicable to certain FRCs. This is understandable, as the constitutive model in MC2020 is not suitable for all FRCs. This is an inevitable result caused by the non-uniformity of fiber types and the randomness of the casting process. However, the results derived in this paper can serve as recommended values for constitutive parameters. If significant errors occur when simulating with the recommended values, adjustments can be made based on specific conditions.

Additionally, the fiber distribution in the 3PBT beams may be different from that in a structure, which may cause different mechanical properties. In that case, all residual stresses should be adjusted, and the influence should be estimated. Further research is required on this issue.

The constitutive model proposed in this paper can serve as a supplement to the MC2020 FRC constitutive models. When selecting a constitutive model, it should be chosen based on the characteristics of the fibers used. The MC2020 constitutive model is more suitable when the fiber pull-out resistance reaches its maximum at a small slippage and the load continues to decrease after the FRC cracks. However, for structural fibers, where the fiber pull-out resistance reaches its maximum at a large slippage, the model proposed in this paper is more appropriate.

## 6. Conclusions

First, this paper shows how the FRC’s constitutive parameters in the *fib* Model Code are derived and explains the differences and reasons behind these between the models in Model Code 2010 and Model Code 2020. Second, it discusses the limitations of the MC2020 FRC constitutive model in application based on the fiber enhancement mechanism in cracked concrete. Third, considering the characteristics of structural FRC, a new post-cracking trilinear constitutive model is proposed as a supplement to the MC2020 constitutive models. The main conclusions are as follows:

1. The stress–strain constitutive models for FRC in the *fib* Model Code are suitable for FRC where the fiber pull-out resistance reaches its maximum at a small slippage and the load continues to decrease after cracking.

2. Although the constitutive models in the *fib* Model Code are suitable for evaluating ULS performance, for structural FRC, where the fiber pull-out resistance reaches its maximum at a large slippage, these models are insufficient to reflect the complex pull-out mechanisms of the fibers and can result in significant errors when evaluating the performance of FRC structures with small cracks. The post-cracking trilinear model proposed in this paper better reflects the fiber pull-out mechanisms and can more accurately simulate the mechanical performance of FRC.

3. The post-cracking trilinear model proposed in this paper is formally similar to the newly proposed trilinear constitutive model in MC2020, but the mechanical background is different. The trilinear constitutive model in MC2020 mainly aims to eliminate numerical singularities and partially considers crack localization. Therefore, the stress at the endpoint of the descending line is related to the tensile strength, and this model is applied when *f*_Fts_ is not less than 0.8*f*_ct_. The trilinear constitutive model proposed in this paper is based on the fiber pull-out mechanism. Thus, the stress at the endpoint of the descending line is related to the residual strength, which is 0.8*f*_Fts_, and this model is applied to structural FRC where the fiber pull-out resistance reaches its maximum at a large slippage.

4. Since the fiber pull-out mechanism is influenced by the fiber shape, diameter, aspect ratio, fiber orientation, and concrete strength, the value of the constitutive parameter proposed in this paper can serve as the recommended value. If significant errors occur when analyzing this parameter, the stress value at the endpoint of the descending line can be adjusted based on the fiber characteristics.

The constitutive models in the *fib* model code perform well when evaluating FRC structure mechanical properties at large crack widths, such as ULS. However, if the main aims are to evaluate the properties at small crack widths, such as SLS, the proposed trilinear model can be more precise.

## Figures and Tables

**Table 1:** Comparison of FRC constitutive models in MC2010 and MC2020.

Type	No.	MC2010	MC2020
Stress-crack opening laws	1	Sketch:	
		Figure 1a	
		Parameter:	
		$f_{Ftu}=\frac{f_{R3}}{3}$	
		Limitation:	
		This law can only be used for evaluating FRC contribution at ULS	
	2	Sketch:	Sketch:
		Figure 1b	Figure 1c
		Parameter:	Parameter:
		$f_{Fts}=0.45f_{R1}$ $f_{Ftu}=f_{Fts}-\frac{w_{u}}{{CMOD}_{3}}(f_{Fts}-0.5f_{R3}+0.2f_{R1})$	$f_{Fts}=0.37f_{R1}$ $f_{Ftu}=f_{Fts}-\frac{w_{u}}{{CMOD}_{3}}(f_{Fts}-0.57f_{R3}+0.26f_{R1})$
		Limitation: This law can only be used for evaluating FRC contribution at ULS	
Stress–strain laws	3	Sketch:	Sketch:
		Figure 2a	Figure 2a
		Parameter:	Parameter:
		$A:\;\sigma_{A}=0.9f_{ct}$ $\;\varepsilon_{A}=\sigma_{A}/E$ $B:\;\sigma_{B}=f_{ct}$ $\;\varepsilon_{B}=0.00015$ C: The intersection point of line BQ and line DE D: The stress is the same as No.2 $\varepsilon_{D}=\varepsilon_{SLS}={CMOD}_{1}/l_{cs}$ E: The stress is the same as No.2 $\varepsilon_{D}=\varepsilon_{\mathrm{ULS}}=\min(\varepsilon_{\mathrm{Fu}},\frac{2.5}{l_{\mathrm{cs}}})$ $Q:\;\sigma_{Q}=0.2f_{ct}$ $\;\varepsilon_{Q}=G_{F}/f_{ct}l_{cs}+\varepsilon_{P}-0.8f_{ct}/E_{c}$ $\;G_{F}=0.073f_{cm}^{0.18}$	$A:\;\sigma_{A}=0.9f_{ct}$ $\;\varepsilon_{A}=\sigma_{A}/E$ $B:\;\sigma_{B}=f_{ct}$ $\;\varepsilon_{B}=0.00015$ C: The intersection point of line BQ and line DE D: The stress is the same as No.2 $\varepsilon_{D}=\varepsilon_{SLS}={CMOD}_{1}/l_{cs}$ E: The stress is the same as No.2 $\varepsilon_{D}=\varepsilon_{ULS}=min(\varepsilon_{Fu},\frac{2.5}{l_{cs}})$ $Q:\;\sigma_{Q}=0.2f_{ct}$ $\;\varepsilon_{Q}=G_{F}/f_{ct}l_{cs}+\varepsilon_{P}-0.8f_{ct}/E_{c}$ $G_{F}=0.085f_{cm}^{0.18}$
		$\varepsilon_{Fu}$: Ultimate strain, where 2% is adopted for structure in bending;	
		$f_{ct}$$:\;\mathrm{tensile}\;\mathrm{strength};\;f_{cm}$$:\;\mathrm{mean}\;\mathrm{compressive}\;\mathrm{strength};\;G_{F}$: fracture energy	
		Limitation:	
		This law can only be used when FRC shows post-cracking softening behavior	
	4	Sketch:	Sketch:
		Figure 2b	Figure 2c
		Parameter:	Parameter:
		The stresses and strains are the same as shown in No.2 and No.3	The stresses and strains are the same as shown in No.2 and No.3, except $C’:\;\sigma_{D}=\beta f_{ct}$$,\;\mathrm{where}\;\mathrm{the}\;\mathrm{stress}\;\mathrm{can}\;\mathrm{be}\;\mathrm{determined}\;\mathrm{by}\;\sigma_{D}$ and line BQ $V:\;\varepsilon_{V}=\alpha \varepsilon_{SLS}$$,\;\mathrm{where}\;\mathrm{the}\;\mathrm{strain}\;\mathrm{can}\;\mathrm{be}\;\mathrm{determined}\;\mathrm{by}\;\varepsilon_{V}$ and line DE
		Limitation:	Limitation:
		$\mathrm{This}\;\mathrm{law}\;\mathrm{should}\;\mathrm{be}\;\mathrm{adopted}\;\mathrm{when}\;f_{Fts}$ $\;\mathrm{is}\;\mathrm{larger}\;\mathrm{than}\;f_{ct}$	$\mathrm{This}\;\mathrm{law}\;\mathrm{should}\;\mathrm{be}\;\mathrm{adopted}\;\mathrm{when}\;f_{Fts}$ $\;\mathrm{is}\;\mathrm{larger}\;\mathrm{than}\;0.8f_{ct}$
	5	Sketch:	Sketch:
		Figure 2d	Figure 2e
		Parameter:	Parameter:
		The stresses and strains are the same as shown in No.2 and No.3	A’: The stress and strain should be determined by tests when the stiffness starts to change $f_{Ft}$: The stress and strain should be determined by uniaxial tests E, F: The equations in No.2 are not valid
		Limitation:	
		This law should be adopted when FRC shows hardening pre-peak behavior	
	6	-	$\mathrm{When}\;\mathrm{the}\;\varepsilon_{P}$(corresponding to the peak stress) exceeds 1%, no softening law is defined, and the constitutive law only reduces to the pre-peak elastic-hardening branch
	7	-	This is recommended for finite element analysis, where the stress–crack width relationship is determined from an inverse analysis by fitting to the force-CMOD * registered in the EN 14651 tests [33]

* CMOD: crack mouth opening displacements; CMOD_1_ equals 0.5 mm, and CMOD_3_ equals 2.5 mm [33].

**Table 2:** Special loads (kN) in three-point bending tests and corresponding fiber contents (kg/m^3^).

No.	F_LOP_	F_min_	F_1_	F_2_	F_3_	F_4_	*C*_f_ *	No.	F_LOP_	F_min_	F_1_	F_2_	F_3_	F_4_	*C*_f_
1	20.2	15.9	16.3	17.4	14.7	11.4	25	19	21.1	16.7	18.3	21.1	15.5	12.2	30
2	20.7	17.0	18.3	20.3	13.7	9.6	25	20	21.2	19.4	21.0	21.4	15.6	10.6	30
3	17.7	11.6	12.0	14.4	12.8	10.9	25	21	18.8	13.0	14.3	15.8	15.4	13.4	30
4	18.8	17.5	21.6	26.0	19.0	13.4	25	22	17.3	15.2	16.3	19.5	18.4	16.8	35
5	19.0	13.9	14.1	16.3	13.8	11.3	25	23	21.1	17.3	19.6	22.7	18.7	16.7	35
6	18.2	16.3	19.3	22.5	14.6	11.1	25	24	18.8	15.7	17.1	18.9	14.6	12.3	35
7	19.7	15.8	18.1	17.1	12.1	9.7	25	25	19.4	16.9	18.7	22.6	20.4	16.5	35
8	17.6	14.4	16.3	20.9	17.0	11.5	25	26	19.3	16.4	17.9	19.5	18.2	17.0	35
9	17.6	15.4	18.4	23.8	17.7	15.2	25	27	20.4	19.1	20.4	21.2	20.0	17.7	35
10	20.0	16.8	18.4	22.0	18.0	14.2	25	28	18.3	17.3	19.2	24.1	24.3	24.3	40
11	18.9	13.4	14.5	17.1	13.3	10.8	25	29	18.2	16.0	18.4	21.5	17.2	14.8	40
12	20.5	18.3	20.8	26.2	19.3	15.5	25	30	17.9	16.5	20.3	26.2	25.0	23.4	40
13	22.7	18.4	21.6	26.9	24.1	20.8	30	31	20.5	19.9	20.6	24.4	23.9	21.4	40
14	20.1	14.7	15.3	16.7	16.5	15.0	30	32	19.3	18.1	20.4	25.3	23.0	20.7	40
15	20.3	16.3	17.9	19.4	14.2	9.0	30	33	13.6	12.7	15.0	19.3	17.4	15.9	40
16	22.0	19.8	23.4	24.6	17.4	15.1	30	34	12.4	11.6	13.8	18.7	16.4	13.4	40
17	15.9	12.9	15.0	17.6	12.9	7.3	30	35	19.7	19.3	21.1	24.4	21.9	19.5	40
18	16.7	13.0	14.2	11.5	5.8	3.8	30								
Avg.	19.1	15.5	17.3	20.3	15.5	12.1	25	Avg.	19.4	16.8	18.3	20.7	18.4	16.2	35
Avg.	19.9	16.0	17.9	19.4	15.3	11.9	30	Avg.	17.5	16.4	18.6	23.0	21.1	19.2	40

* *C*_f_: the fiber content.

**Table 3:** The correlation coefficients of F_min_ and other special loads.

F_min_-F_LOP_	F_min_-F_1_	F_min_-F_2_	F_min_-F_3_	F_min_-F_4_
0.57	0.7	0.67	0.52	0.42

**Table 4:** Stress values of point C’ for parameter analysis.

Law	Bilinear Law	Trilinear Law			
Symbol	Bi-L	Tri-1	Tri-2	Tri-3	Tri-4
Stress at point C’ (σ_c_’)	2.218	1.948	1.702	1.455	1.209

**Table 5:** The simulation errors of F_min_ and F1 caused by different σ_c_’ values.

	Test Data	Bi-L	Tri-1	Tri-2	Tri-3	Tri-4	Optimal
F_min_	15.52	-	17.1	15.9	14.6	13.3	15.6
error	-	-	10%	2%	−6%	−14%	1%
F_1_	17.35	19.6	18.7	17.7	16.9	16.1	17.5
error	-	13%	8%	2%	−3%	−7%	1%

**Table 6:** Model parameters for different SFRC classifications (MPa).

	Test 1	Test 2	Test 3	Test 4	Test 5	Test 6
*f^f^*_ct,L_	5.64	6.02	6.46	6.61	7.00	5.92
*f*_R1_	3.83	4.63	5.23	6.59	7.46	7.67
*f*_R3_	4.11	4.24	4.71	6.48	5.55	8.54
*f*_ct_	3.32	3.54	3.80	3.89	4.12	3.48
*f*_Fts_	1.42	1.71	1.94	2.44	2.76	2.84
*f*_Ftu_	1.35	1.21	1.32	1.98	1.22	2.87

**Figure A1:**
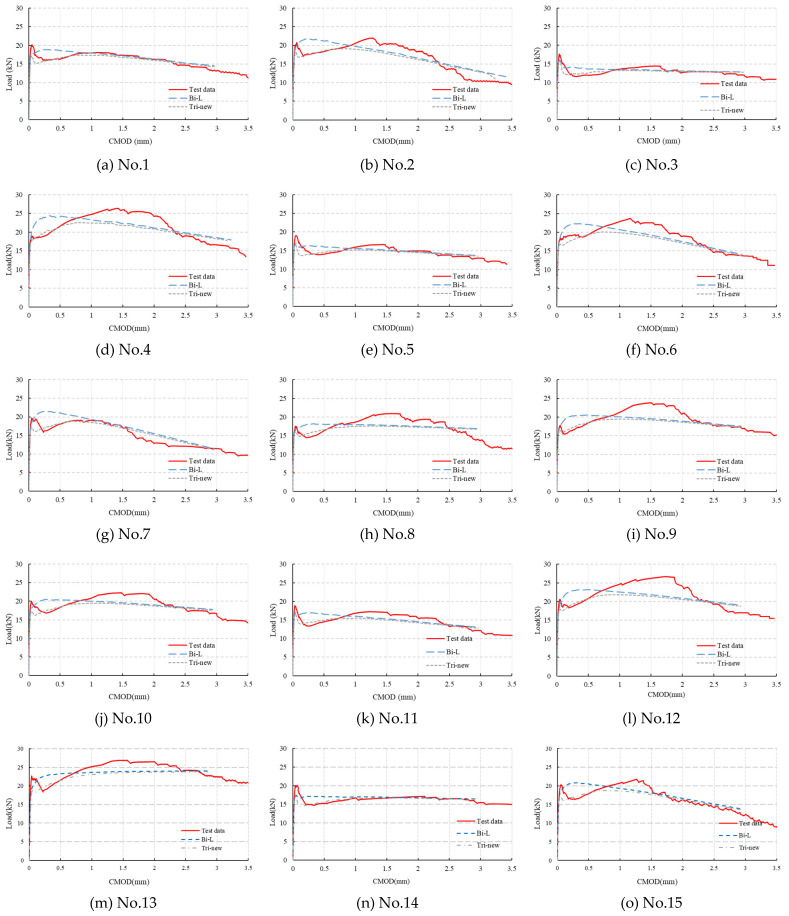

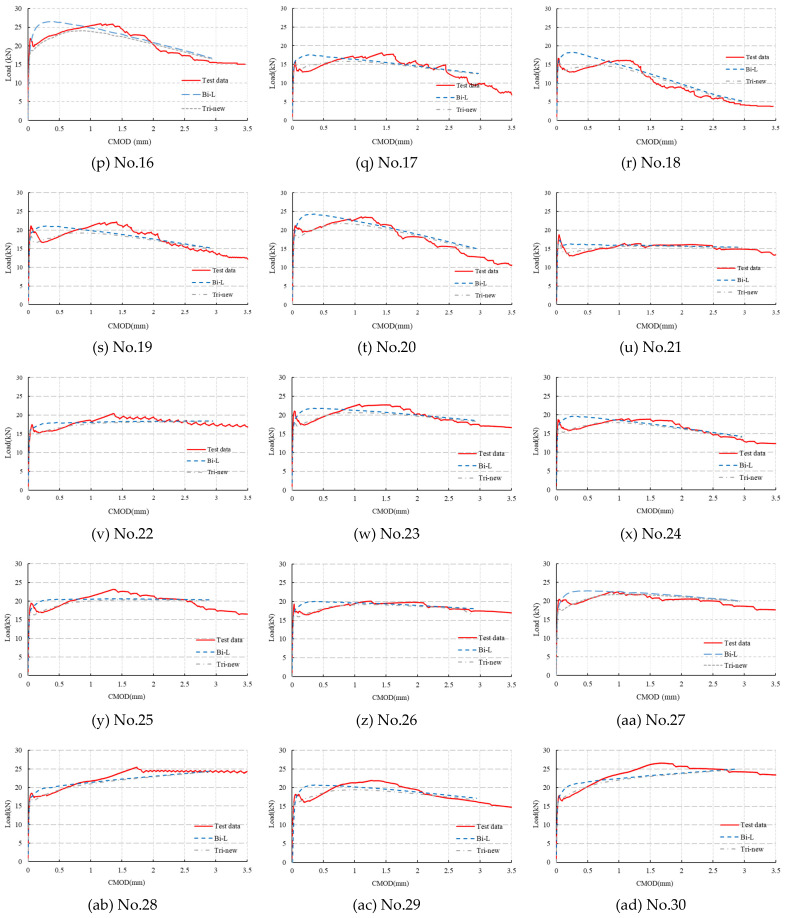

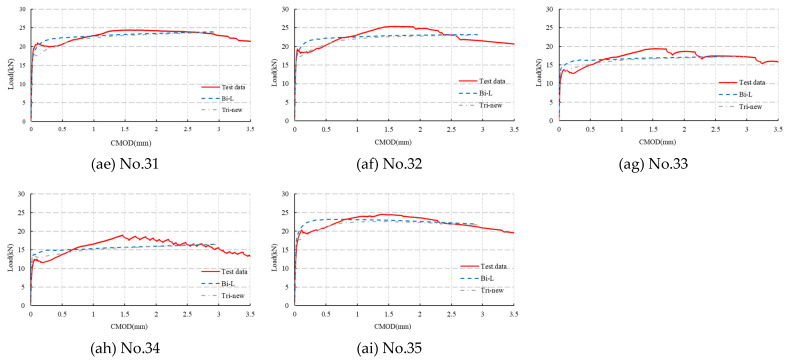
Comparison of simulation results through different constitutive laws.

## Data Availability

The original contributions presented in this study are included in the article; further inquiries can be directed to the corresponding author.

## Appendix A

All 35 3PBTs shown in Table 2 were simulated with the post-cracking bilinear model and proposed trilinear model. The simulation results are shown in Figure A1. The subgraph caption for each figure in Figure A1 relates to the data shown in Table 2, for example, No.1 in Figure A1 relates to the first set of data shown in Table 2.
